# Optical genome mapping of a complex structural rearrangement family line on chromosome 18

**DOI:** 10.1186/s41065-025-00602-5

**Published:** 2025-12-02

**Authors:** Liyi Cai, Yuying Jiang, Na Zhang, Xinying Chen

**Affiliations:** Prenatal Diagnosis Center, Quanzhou Women’s and Children’s Hospital, Quanzhou, Fujian Province 362000 China

**Keywords:** Prenatal diagnosis, Complex abnormality, Optical genome mapping, Chromosome 18

## Abstract

**Background:**

Complex chromosomal rearrangement (CCR) refers to a structural rearrangement involving at least two chromosomes or a minimum of three breakpoints. CCR may lead to intellectual disability, structural anomalies, infertility, and recurrent miscarriages. Chromosome karyotyping and chromosomal microarray analysis (CMA) are unable to detect complex chromosomal rearrangements. As multiple diagnostic approaches are available in clinical practice for detecting chromosomal structural abnormalities and copy number variations—each with its own advantages and limitations—selecting the appropriate testing method is crucial for effective clinical management. Optical genome mapping (OGM) is an advanced genomic technology that utilizes ultra-long single-molecule analysis to comprehensively detect chromosomal aberrations and structural variants at high resolution.

**Material and methods:**

Amniocentesis was performed for a 36-year-old multipara (advanced maternal age), with subsequent comprehensive fetal genetic analysis including chromosome karyotyping, CMA, and OGM. Family members underwent peripheral blood karyotyping and OGM.

**Results:**

The fetal karyotype derived from amniotic fluid was 46,XN,?ins(18)(q21.2;p11.31p11.2). CMA demonstrated duplications of four segments and a deletion of one segment on chromosome 18. Therefore, OGM was performed on the fetal and family members to further elucidate the chromosomal structure. The fetus has derived CCRs on chromosome 18 of maternal origin. In contrast, both the mother and the second daughter, who carried the identical CCRs, were phenotypically normal.

**Conclusion:**

OGM is of significant importance in the diagnosis and characterization of CCRs.

OGM plays a critical role in diagnosing complex chromosomal rearrangements and has proven to be invaluable in clinical utility.

## Introduction

CCR involves structural rearrangements of chromosomes, affecting at least two chromosomes or involving at least three breakpoints [1]. Such rearrangements often result in congenital anomalies, including infertility, recurrent miscarriage, and intellectual disability. CCRs are classified based on the localization and distribution of breakpoints, either with intrachromosomal rearrangement (insertions, inversions, duplications) or without. Chromosome karyotype analysis can detect numerical and structural chromosome abnormalities but is limited to abnormalities larger than 10 Mb [2]. CMA cannot detect inversions, balanced translocations, or other variants that do not alter chromosome copy numbers [3]. Fluorescence in situ hybridization (FISH) can detect deletions, duplications, and balanced translocations of smaller fragments but requires prior knowledge of the fragments to be detected and corresponding probe preparation, limiting its throughput [4]. Each test has its strengths and limitations. However, even when used in combination, a significant proportion of fetal genetic disorders remain undiagnosed, highlighting the need for more accurate and convenient testing methods.

OGM does not require cell culture and can rapidly generate high-resolution genome-wide restriction endonuclease maps. It can distinguish structural variations as small as 500 bp, detecting cryptic balanced translocations or small structural variations. OGM can also identify complex structural variations, including insertions, deletions, inversions, and translocations, determining the position and direction of variant fragments, refining breakpoints, and discovering gene fusions.

This study aims to identify the true structural variants of fetal chromosomes and their origin using OGM, guiding prenatal diagnosis.

## Material and methods

### Participants

**Fig. 1:**
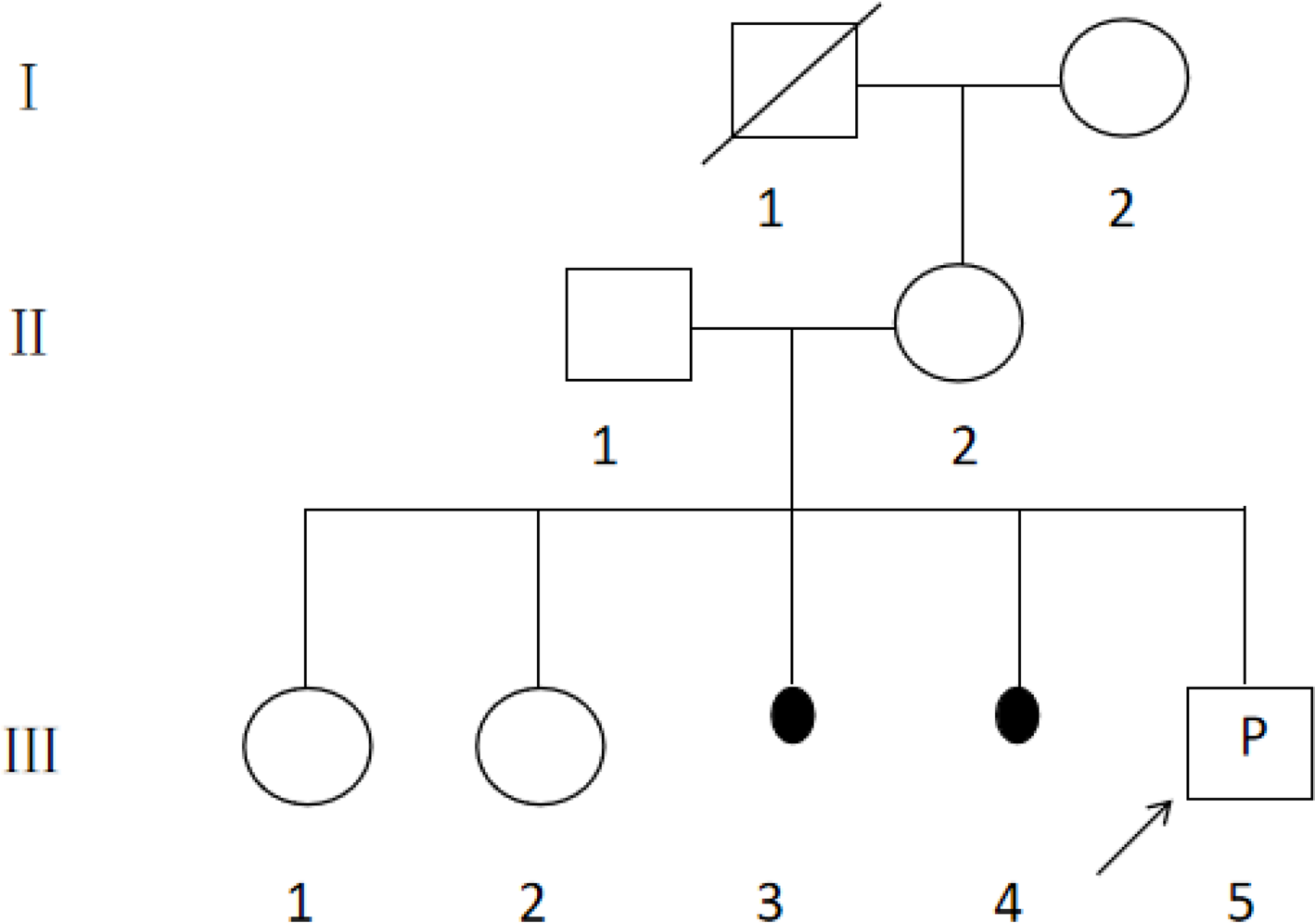
Family pedigree

A 36-year-old gravida 5, para 2 pregnant woman (Fig. 1, Ⅱ−2) from Quanzhou, Fujian Province, China, was referred to the Prenatal Diagnosis Center of Quanzhou Women’s and Children’s Hospital at 23^+3^ weeks of gestation. She is healthy, with a singleton pregnancy, conceived spontaneously, and no special conditions during pregnancy. The couple (Fig. 1, Ⅱ−1 and Ⅱ−2) denied consanguineous marriage or any related inherited history. She has two daughters (Fig. 1, Ⅲ−1 and Ⅲ−2), aged 13 and 5 years, both with normal phenotypes. Her third pregnancy ended in a spontaneous abortion (Fig. 1, Ⅲ−3), and her fourth in a medication abortion (Fig. 1, Ⅲ−4). During the routine second-trimester ultrasound at 23^+2^ weeks of gestation, two soft markers were noted: (1) echogenic intracardiac focus (EIF) in the fetal left ventricle, and (2) mild left fetal pyelectasis (0.54 cm).

Amniocentesis was performed at 23^+3^ weeks for fetal amniotic fluid karyotyping and CMA due to advanced maternal age and fetal ultrasound soft markers. Following genetic counseling and informed consent, the fetus underwent chromosome karyotype analysis and CMA to detect chromosomal abnormalities and copy number variants (CNVs). Results indicated fetal chromosome 18 abnormalities, with microduplications in four segments and a microdeletion in one segment. To determine the true structural variations and the origin of fetal chromosomes, chromosomal karyotyping and OGM were performed on the family line.

### Karyotype analysis

Chromosome karyotyping followed the procedure in our prenatal diagnosis center's laboratory [5]. Fetal amniotic fluid (20 mL) was cultured for 7–10 days, and cells were harvested using the tryptic digest method. Peripheral blood samples (2 mL) from family members were collected for routine lymphocyte culture. Chromosomes were prepared using the Sinochrome ChromprepII automatic chromosome harvesting system (Shanghai Lechen Biotechnology Co., Ltd.) and karyotyped by G-banding. Amniotic fluid cells were analyzed for 30 karyotypes per case, and peripheral blood for 20 karyotypes per case, with 5 karyotypes analyzed. Karyotype descriptions followed the International System for the Nomenclature of Human Cytogenetics (ISCN2020) [6].

### CMA analysis

Genomic DNA was extracted from enrolled subjects using the QIAamp DNA Blood Kit (QIAGEN, Germany), with reference to the manufacturer's protocol [7], (www.qiagen.com). Following hybridization, the microarrays were washed and stained according to the standard protocol. Scanning was then performed using a GeneChip scanner. (Affymetrix Cytocan 750 K GeneChip Kit, USA). Fluorescence signals were analyzed with Chromosome Analysis Suite (ChAS) v4.0 software. Copy number variants (CNVs) pathogenicity was interpreted according to the American College of Medical Genetics (ACMG) standards [8]. Reference resources included the Database of Genomic Variants (DGV), Online Mendelian Inheritance in Man (OMIM), DECIPHER, PubMed, and other relevant databases for data analysis and interpretation.

### OGM analysis

Patient blood DNA and fetal amniotic fluid DNA were extracted using the Bionano Prep SP Blood and Cell DNA Isolation Kit V1 (Bionano Genomics, USA) according to the Bionano Prep Blood DNA Isolation Protocol. Quantification was performed with the Qubit® BR (Broad Range) dsDNA Assay Kit, with DNA concentrations ranging from 36 to 150 ng/μL. Sequence-specific labeling was conducted using the Bionano Prep DLS Labeling Kit, identifying the 6-base sequence pattern (CTTAAG motif) and labeling it with green fluorescence. The entire DNA molecule backbone was counterstained blue, resulting in fragments of blue DNA molecules with green fluorescent signals. After labeling, DNA was quantified with the Qubit dsDNA HS (High Sensitivity) Assay Kit and Qubit 3.0 Fluorometer (ThermoFisher Scientific, USA) to achieve a concentration suitable for mounting. The labeled DNA was added to the Saphyr chip, inserted into the Saphyr instrument, and run. Following data generation, quality control was performed using Access Analytics software. The data met the following quality thresholds: Avg N50 > 230 kb, Avg Label Density per 100 kb: 14 ~ 17, Avg Map Rate > 70%, Avg PLV < 10%, Avg NLV < 15%.

## Results

### Results of chromosome karyotyping

**Fig. 2:**
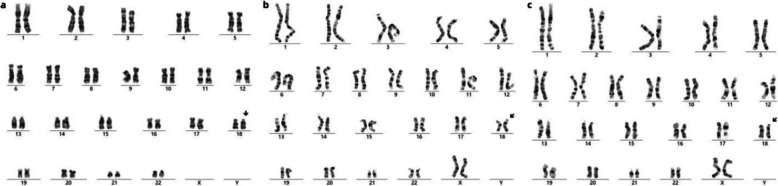
Results of karyotype analysis (**a.** the fetus (Fig. 1, Ⅲ−5; **b**. the mother (Fig. 1, Ⅱ−2); **c**. the sister (Fig. 1, Ⅲ−2))

The chromosomal karyotypes of the fetus (Fig. 1, Ⅲ−5) and his second sister (Fig. 1, Ⅲ−2) revealed a structural abnormality of chromosome 18. This rearrangement was characterized by an inverted insertion of the 18p11.3p11.2 segment into the 18q21.2 region. Both offsprings inherited this rearrangement from their mother (Fig. 2), who, despite carrying the abnormality, presented no clinical phenotype. In contrast, karyotype analyses of the father, the eldest sister (Fig. 1, Ⅲ−1) and the grandmother (Fig. 1, Ⅰ−2) yielded normal results, with no chromosomal abnormalities detected (Table 1).

**Table 1:** Chromosomal segments involved in this case

Chromosome Region	Physical Location (GRCh38)	Size (Mb)	Copy Number	Genes	DECIPHER	Clinical Significance	DGV	Mother(Ⅱ−2)	Fetus (Ⅲ−5)
18p11.31	3,195,545–3,642,578	0.45	3	*TGIF1,MYOM1,MYL12A,DLGAP1*	No evidence available	VUS	Cases with larger duplications reported as non-penetrant (e.g., 236920__DUP, 236243__DUP)	+	+
18q12.1q12.2	34,948,475–35,774,573	0.83	3	*MAPRE2,ZNF397,INO80C,GALNT1*	No evidence available	VUS	No known polymorphisms larger than this duplication	+	+
18q21.1	46,305,911–46655302	0.35	3	*LOXHD1,ARK2C*	No evidence available	VUS	No known polymorphisms larger than this duplication	+	-
18q21.33q22.1	63,221,093–63966622	0.75	3	*SERPINB7,KDSR,BCL2,VPS4B,SERPINB5*	No evidence available	VUS	Cases with larger duplications reported as non-penetrant (e.g., 243967__DUP, 244183__DUP)	+	+
18q22.1	67,857,144–68,313,028	0.46	3	-	No evidence available	LB	Cases with larger duplications reported as non-penetrant (e.g., 244805__DUP, 244769__DUP, 243967__DUP)	+	-
18q22.3	74,219,503–74870378	0.65	3	*ZNF407,CYB5A,C18orf63,DIPK1C,CNDP2*	No evidence available	VUS	Cases with larger duplications reported as non-penetrant (e.g., 245192__DUP, 243967__DUP)	+	+
18q21.1	46,655,302–48560072	1.9	1	*SMAD2,LOXHD1,IER3IP1,ST8SIA5,PIAS2*	No evidence available	VUS	No known polymorphisms larger than this deletion	-	+
7p21.2	16282707_16363650	0.0534	1	*CRPPA*	No evidence available	VUS	Cases with larger deletions reported as non-penetrant (e.g., 464934__DEL, 464928__DEL, 464935__DEL)	+	-

### Results of CMA

**Fig. 3:**
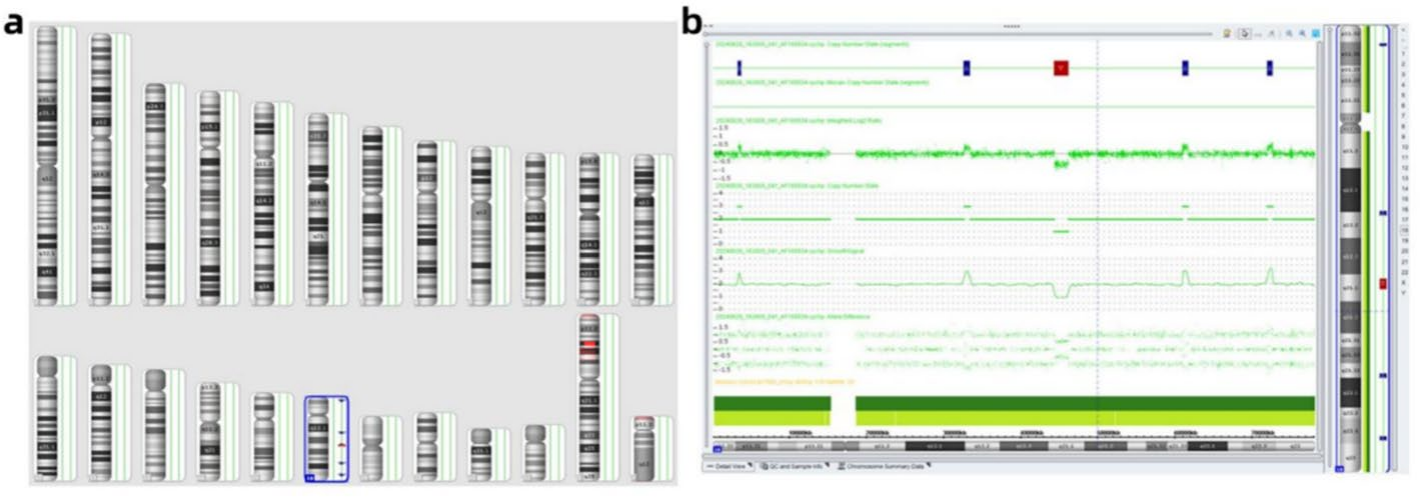
CMA result of the fetus (Fig. 1, Ⅲ−5) (**a** chromosome simulation karyotype diagram; **b** detail of chromosome 18 deletions and duplications)

In the fetus, CMA identified four duplications and one deletion on chromosome 18. The duplications were located at 18p11.31 (0.43 Mb;3209776_3644083), 18q12.1q12.2 (0.82 Mb;32542816_33368516), 18q21.33q22.1 (0.74 Mb; 60880283_61625358), and 18q22.3 (0.71 Mb; 71876693_72589282). The 1.85-Mb deletion was located at 18q21.1 (44240401_46091852) and encompassed 10 protein-coding genes, including *SMAD2* (OMIM: 601,366). Based on the DECIPHER and gnomAD databases, *SMAD2* is predicted to be a haplo insufficient gene. According to the ACMG standards and guidelines, all CNVs identified in the fetus were classified as variants of uncertain significance (VUS) (Fig. 3).

### Results of OGM

OGM analysis of the mother and her second daughter (Fig. 1, Ⅲ−2) revealed identical CCRs. The CCRs consisted of six duplications on chromosome 18, located at 18p11.31 (0.44 Mb; 3195545_3642578), 18q12.1q12.2 (0.82 Mb; 34948475_35774573), 18q21.1 (0.34 Mb; 46305911_46655302), 18q21.33q22.1 (0.74 Mb; 63221093_63966622), 18q22.1 (0.45 Mb; 67857144_68313028), and 18q22.3 (0.65 Mb; 74219503_74870378). Additionally, a 53.4-kb deletion was identified at 7p21.2 (16282707_16363650). According to ISCN2020, this complex rearrangement was designated as: 46,XX,del(7)(p21.2p21.2),der(18)(18pter → p11.31::p11.21q21.1::q22.3q22.3::q21.1q21.1::p11.21p11.31::q22.1q21.33:q22.3q22.3::q12.1q12.1::q21.1 → 18qter).

**Fig. 4:**
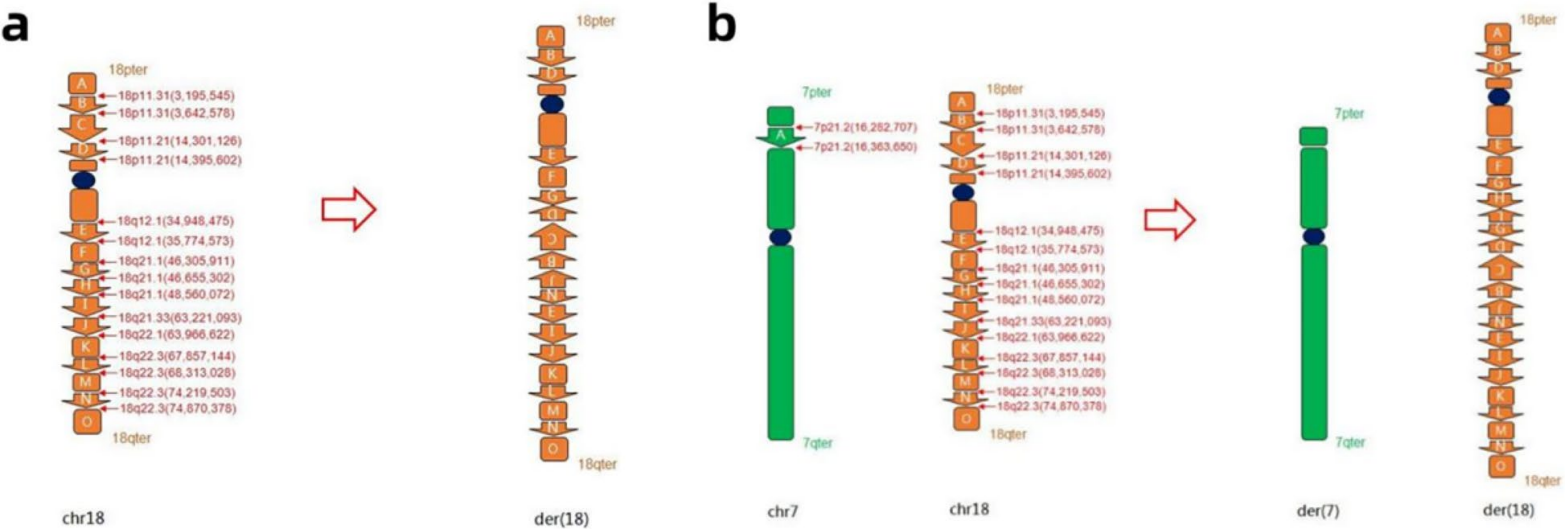
Rearrangement pattern of abnormal chromosome structure (**a** the fetus (Fig. 1, Ⅲ−5); **b** the mother (Fig. 1, Ⅱ−2)

OGM confirmed the presence of the CNVs on fetal chromosome 18 (four duplications and one deletion), with genomic ranges that were largely consistent with the CMA results. Furthermore, OGM analysis suggested a CCR. Based on the OGM findings, the fetal karyotype was designated as 46,XN,rec(18)(18pter → p11.31::p11.21q21.1::p11.21p11.31::q22.1q21.33::q22.3q22.3::q12.1q12.1::q21.1 → 18qter)dmat (Fig. 4).

No clinically significant structural variants were detected by OGM in the father or the eldest sister.

### Pregnancy outcome

**Fig. 5:**
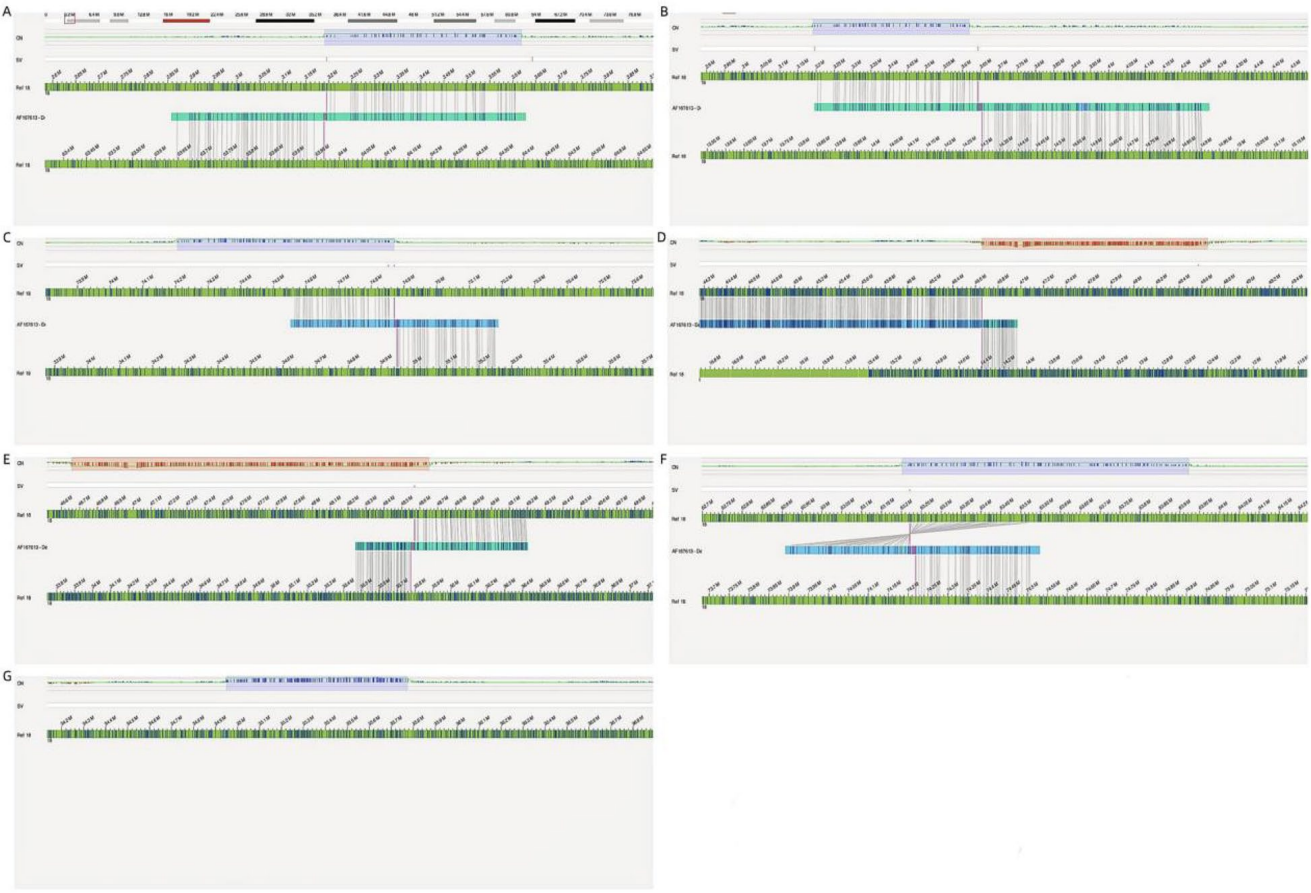
Detailed diagram of fetal chromosome variants. (**A**:ogm[GRCh38] fus(18;18)(p11.31;q22.1)(3,195,545;63,966,622); **B**:ogm[GRCh38] fus(18;18)(p11.31;p11.21)(3,642,578;14,301,126); **C**:ogm[GRCh38] fus(18;18)(q22.3;q12.1)(74,870,378;34,948,475); **D**:ogm[GRCh38] fus(18;18)(q21.1;p11.21)(46,655,302;14,395,602); **E**:ogm[GRCh38] fus(18;18)(q21.1;q12.2)(48,570,712;35,774,573); **F**:ogm[GRCh38] fus(18;18)(q21.33;q22.3)(63,221,093;74,219,503); **G**:ogm[GRCh38] 18q12.1q12.2(34948475_35774573) × 3)

**Fig. 6:**
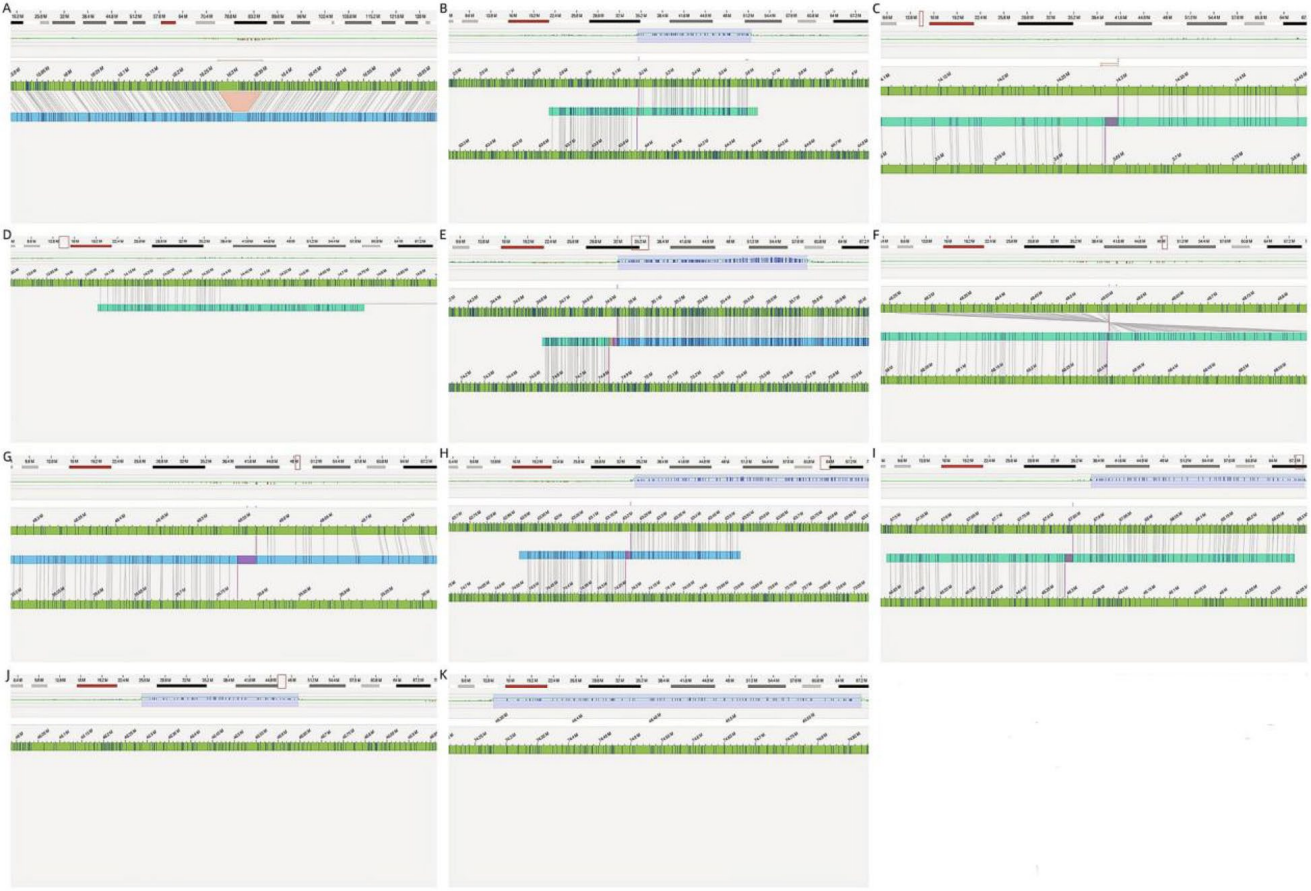
Detailed diagram of mother’s chromosome variants. (**A** :ogm[GRCh38] 7p21.2(16282707_16363650) × 1; **B** :ogm[GRCh38] fus(18;18)(p11.31;q22.1)(3,195,545;63,966,622); **C **:ogm[GRCh38] fus(18;18)(p11.21;p11.31)(14,301,126;3,642,578); **D **:ogm[GRCh38] fus(18;18)((p11.21;q21.1)(14,395,602;46,655,302); **E **:ogm[GRCh38] fus(18;18)(q12.1;q22.3)(34,948,475;74,870,378); **F **:ogm[GRCh38] fus(18;18)(q21.1;q22.1)(48,560,072;68,313,028); **G **:ogm[GRCh38] fus(18;18)(q21.1;q12.2)(48,570,712;35,774,573); **H **:ogm[GRCh38] fus(18;18)(q21.33;q22.3)(63,221,093;74,219,503); **I **:ogm[GRCh38] fus(18;18)(q22.1;q21.1)(67,857,144;46,305,911);** J **:ogm[GRCh38] 18q21.1(46305911_46655302) × 3; **K **:ogm[GRCh38] 18q22.3(74219503_74870378) × 3)

The ultrasound examination of the fetus during pregnancy did not show any significant abnormality, and the couple chose to continue the pregnancy with informed consent, delivering a baby boy at full term without any apparent abnormalities after birth (Figs. 5, 6).

## Discussion

CCR carriers can exhibit a range of phenotypes, including phenotypically normal, male infertility, intellectual disability, and/or congenital malformations. It has been reported that approximately 70% of CCR carriers are phenotypically normal, 20–25% have congenital malformations and/or intellectual disability and an additional 5–10% are identified during prenatal diagnosis [9]. The normalization of the phenotype of CCR carriers depends mainly on whether the chromosomes are balanced or whether the chromosomal structural rearrangement breakpoints disrupt genes that are important for growth and development. Given the challenge of predicting phenotypes in prenatal diagnostic cases, it is crucial to select the most appropriate testing strategy to accurately delineate the chromosomal structure. This is essential for facilitating informed genetic counseling and prognostic assessment. OGM testing is an emerging technology that can sensitively identify insertions, deletions, translocations, and inversions [10]. Previous findings have indicated that OGM technology is advantageous in patients with complex chromosomal rearrangements or questionable karyotype results [11, 12]. Xie M et al. [13] reported that among OGM, CMA, and karyotyping, OGM demonstrated the highest diagnostic yield (25%) in a study of 204 amniotic fluid samples. The combination of OGM and karyotyping achieved a diagnostic yield of 29.41%. Notably, the addition of CMA to this combination (i.e., OGM + CMA + chromosome karyotype analysis) did not further increase the diagnostic rate, which remained at 29.41%. This result indicates that combining all three technologies provides no additional diagnostic benefit over the combination of OGM and karyotyping alone. Therefore, the two-method approach (OGM + chromosome karyotype analysis) is fully sufficient to achieve the maximum detection efficacy observed in this study.

Carriers of balanced chromosomal insertions are usually phenotypically normal. However, during meiosis, the rearranged chromosomes can lead to a risk (approximately 15%) of producing gametes with unbalanced genetic material [14]. The majority of these aberrant gametes result in deletions or duplications of the inserted fragment itself, while a smaller proportion lead to unbalanced rearrangements involving non-inserted chromosomal segments [15]. These conditions often lead to problems such as spontaneous abortions, stillbirths, and fetal anomalies. In this case, the spontaneous abortion experienced by the mother could be attributed to the conception of an unbalanced embryo resulting from her CCRs. During meiosis, the normal chromosome and the chromosome with the insertion form a characteristic loop structure (pairing loop) to facilitate homologous pairing. The length of the inserted chromosomal segment influences the configuration of meiotic pairing. Shorter inserts may pair without forming a stable loop structure, leading to crossover events that produce four gamete types: normal, balanced carrier, and those with partial monosomy or partial trisomy for the relevant segments. Long insertion fragments can form double loops that pair in complete association to form abnormal gametes with double or no filaments [14]. In this study, the chromosomal insertion segments accounted for approximately 14% of the chromosome's length. Given their relatively small size, we hypothesized that crossovers would frequently occur within the pairing loop during meiosis, leading to genetic observations consistent with incomplete linkage [16]. OGM validated the presence of a CCR in the mother and her two offspring (Ⅲ−2, Ⅲ−5). This rearrangement was characterized by an inverted insertion of an approximately 11.2-Mb segment from 18p11.31p11.21 (chr18: 31,95,545–46,55,302) into the 18q21.1 region. OGM precisely delineated the breakpoints at both the donor and insertion sites.

Their offspring may develop 18p deletion syndrome or 18p duplication syndrome. 18p deletion syndrome is a chromosomal disorder caused by total or partial deletion of the short arm of chromosome 18, with a prevalence of approximately 1:50,000 in live births [17]. And the clinical phenotypes are diverse and atypical, depending on the size of the missing fragments and the location of the breakpoints [18]. Meanwhile there are fewer reports of patients with 18p duplication, with clinical phenotypes ranging from normal, mildly abnormal to varying degrees of intellectual disability [19, 20]. We revealed a complex inheritance pattern. During meiosis, an unbalanced segregation event occurred, resulting in the fetus inheriting a derivative chromosome 18 with a structure that differs from the mother's rearrangement. This complexity posed a significant challenge for prenatal genetic counseling. Furthermore, the breakpoints of this rearrangement in the affected individuals (Fig. 1, Ⅱ−2, Ⅲ−2, Ⅲ−5) were mapped to regions containing the *LOXHD1* (OMIM: 613,072) and *SERPINB7* (OMIM: 603,357) genes, suggesting a potential contribution to the phenotypic outcomes. The lack of evidence for dosage sensitivity in the ClinGen database for the genes involved at the breakpoints (*LOXHD1*, *SERPINB7*), together with the normal phenotype observed in the mother and sister (Ⅲ−2), provides reassuring evidence that the disruption of these genes by the breakpoints may pose a low risk of causing severe pathogenic effects in the fetus.

Whether CCR causes clinical abnormalities depends on three factors: affected protein-coding genes at breakpoints, associated copy number changes, and the clinical significance of these genomic alterations. A key question for prenatal diagnosis is whether the deleted segments will cause a clinical phenotype, given that the duplicated segments were inherited from unaffected family members and are thus likely benign. The deleted region contains 11 RefSeq protein-coding genes. Notable genes among these include *IER3IP1* (OMIM: 609,382), *LOXHD1* (OMIM: 613,072), *SMAD2* (OMIM: 601,366), *ST8SIA5* (OMIM: 607,162), and *PIAS2* (OMIM: 603,567). The disease database DECIPHER has not reported any pathogenic cases of similar deletion, and the population databases DGV and gnomAD have not reported any polymorphisms larger than this deletion, so the clinical significance of this deletion is not clear. Heterozygous mutations in *SMAD2* cause two major autosomal dominant disorders: congenital heart disease-8, with or without heterotaxy (CHTD8), and Loeys-Dietz syndrome-6 (LDS6). Previous studies have shown that pathogenic heterozygous mutations in the *SMAD2* gene are implicated in distinct clinical presentations: they are a recognized cause of congenital heart defects (CHTD8) and are also associated with aneurysmal disease [21, 22].

Since prenatal ultrasound showed no structural abnormalities and fetal growth was appropriate for gestational age, the family opted to continue the pregnancy. A healthy male infant was delivered, passing newborn hearing screening. Long-term phenotypic follow-up is recommended. Although the OGM shows its high resolution and accuracy, it still has its limitations. In addition to its inability to detect Robertsonian translocations, OGM also has difficulty characterizing highly repetitive regions, chromosomal telomeric regions, and heterochromatic regions [12, 23, 24].

The fetus, mother, and second sister all share CCRs and abnormal CNVs involving chromosome 18. Nevertheless, they are all phenotypically normal. Potential explanations for this discordance include: first, although the breakpoints of the CCRs involved the protein-coding genes *LOXHD1* (OMIM: 613,072) and *SERPINB7* (OMIM: 603,357), which are associated with pathogenic variants, the breakpoints did not disrupt the structure of these genes. It is also possible that the CCRs disrupted gene function. Given the autosomal recessive inheritance of both genes and the presence of wild-type alleles, such a disruption would not be expected to cause an abnormal phenotype in carriers. Furthermore, despite the presence of CNVs affecting multiple segments in this case, the specific genomic regions involved are not known to cause clinical symptoms when duplicated or deleted.

In this study, OGM was employed to characterize a fetal chromosome 18 complex structural rearrangement inherited from the phenotypically unaffected mother who carried an identical rearrangement. OGM's unique capacity to precisely identify breakpoints and implicated genes proves particularly valuable for elucidating complex chromosomal abnormalities.This approach plays a pivotal role in the clinical work of providing genetic counseling, prenatal diagnosis, fertility risk assessment, and assisted reproductive technology.

## Data Availability

No datasets were generated or analysed during the current study.

## Abbreviations

OGM: Optical genome mapping
CMA: Chromosome microarray analysis
CCR: Complex chromosomal rearrangement
EIF: Echogenic intracardiac focus
FISH: Fluorescence in situ hybridization
CNVs: Copy number variants
ACMG: American college of medical genetics
DGV: Database of genomic variants
OMIM: Online mendelian inheritance in man
VUS: Variants of uncertain significance
LB: Likely benign

## References

[1] Madan K. What is a complex chromosome rearrangement? Am J Med Genet A. 2013;161A(5):1181–4. 23532917 10.1002/ajmg.a.35834

[2] Rao H, Liu Y, Lu Q, Huang N, Zhou J. The value of combined use of chromosomal karyotyping and chromosome microarray analysis for prenatal diagnosis. Chin J Med Genet. 2020;37(4):392–6. 10.3760/cma.j.issn.1003-9406.2020.04.007 32219820

[3] Ronan A. Chromosome microarray analysis: a soothing guide. J Paediatr Child Health. 2018;54(6):599–601. 29573507 10.1111/jpc.13869

[4] Gozzetti A, Le Beau MM. Fluorescence in situ hybridization: uses and limitations. Semin Hematol. 2000;37(4):320–33. 11071355 10.1016/s0037-1963(00)90013-1

[5] Zhuang J, Wang Y, Zeng S, Lv C, Lin Y, Jiang Y. A prenatal diagnosis and genetics study of five pedigrees in the Chinese population with Xp22.31 microduplication. Mol Cytogenet. 2019;11(12):50. 10.1186/s13039-019-0461-1 PMC6907354 31857824

[6] McGowan-Jordan J, Ros H, Sarah M. ISCN 2020: an international system for human cytogenomic nomenclature (2020). Basel: Karger; 2020.

[7] Chen X, Jiang Y, Zeng S, Zhuang J, Lin N. Prenatal diagnosis of fetuses with absent/hypoplastic nasal bone in second-trimester using chromosomal microarray analysis. Birth Defects Res. 2024;116(5):e2351. 38766695 10.1002/bdr2.2351

[8] Kearney, H. M., Thorland, E. C., Brown, K. K., Quintero-Rivera, F., South, S. T., & Working Group of the American College of Medical Genetics Laboratory Quality Assurance Committee. American College of Medical Genetics standards and guidelines for interpretation and reporting of postnatal constitutional copy number variants. Genet Med. 2011;13(7):680–5. 21681106 10.1097/GIM.0b013e3182217a3a

[9] Pellestor F, Anahory T, Lefort G, Puechberty J, Liehr T, Hédon B, et al. Complex chromosomal rearrangements: origin and meiotic behavior. Hum Reprod Update. 2011;17(4):476–94. 21486858 10.1093/humupd/dmr010

[10] Sahajpal NS, Barseghyan H, Kolhe R, Hastie A, Chaubey A. Optical genome mapping as a next-generation cytogenomic tool for detection of structural and copy number variations for prenatal genomic analyses. Genes. 2021;12(3):398. 33799648 10.3390/genes12030398 PMC8001299

[11] Wang H, Yang Y, Yang N, Wang Y, Li H, Hu W. Optical genome mapping analysis of a Chinese pedigree with a rare chromosome 17 paracentric inversion insertion. Zhonghua Yi Xue Yi Chuan Xue Za Zhi. 2023;40(6):727–32. 37212011 10.3760/cma.j.cn511374-20220107-00012

[12] He SJ, Zhang ZQ, Huang YJ, Xu LN, Chen YQ, Fang C, et al. Application of optical genome mapping technology in detecting complex chromosomal rearrangement. J Sun Yat-Sen University (Medical Sciences). 2023;44(6):943–8.

[13] Xie M, Zheng ZJ, Zhou Y, Zhang YX, Li Q, Tian LY, et al. Prospective investigation of optical genome mapping for prenatal genetic diagnosis. Clin Chem. 2024;70(6):820–9. 38517460 10.1093/clinchem/hvae031

[14] Madan K, Menko FH. Intrachromosomal insertions: a case report and a review. Hum Genet. 1992;89(1):1–9. 1577455 10.1007/BF00207032

[15] Ardalan A, Prieur M, Choiset A, et al. Intrachromosomal insertion mimicking a pericentric inversion: molecular cytogenetic characterization of a three break rearrangement of chromosome20. Am J Med Genet A. 2005;138A(3):288–93. 16158442 10.1002/ajmg.a.30966

[16] Bhatt S, Moradkhani K, Mrasek K, et al. Breakpoint characterization: a new approach for segregation analysis of paracentric inversion in human sperm JJ. Mol Hum Reprod. 2007;13(10):751–6. 17913851 10.1093/molehr/gam048

[17] Turleau C. Monosomy 18p. Orphanet J Rare Dis. 2008;19(3):4. 10.1186/1750-1172-3-4 PMC2265258 18284672

[18] Jin Q, Qiang R, Cai B, Wang X, Cai N, Zhen S, et al. The genotype and phenotype of chromosome 18p deletion syndrome: case series. Medicine (Baltimore). 2021;100(18):e25777. 33950970 10.1097/MD.0000000000025777 PMC8104293

[19] Kariminejad A, Kariminejad R, Moshtagh A, et al. Pericentric inversion of chromosome 18 in parents leading to a phenotypically normal child with segmental uniparental disomy 18. Eur J Hum Genet. 2011;19(5):555–60. 21326286 10.1038/ejhg.2010.252 PMC3083628

[20] Nannan Y, LiYan Z, Xin J, et al. Zhonghua Yi Xue Yi Chuan Xue Za Zhi. 2022;39(01):121–2.

[21] Zaidi S, Choi M, Wakimoto H, Ma L, Jiang J, Overton JD, et al. De novo mutations in histone-modifying genes in congenital heart disease. Nature. 2013;498(7453):220–3. 23665959 10.1038/nature12141 PMC3706629

[22] Micha D, Guo DC, Hilhorst-Hofstee Y, van Kooten F, Atmaja D, Overwater E, et al. SMAD2 mutations are associated with arterial aneurysms and dissections. Hum Mutat. 2015;36(12):1145–9. 26247899 10.1002/humu.22854

[23] Zhang Z, He S, Li X, Cheng K, Wei Y, Ren Z. Application of optical genome mapping technology for the detection of chromosomal structural variations. Zhonghua Yi Xue Yi Chuan Xue Za Zhi. 2024;41(3):257–65. 38448011 10.3760/cma.j.cn511374-20230107-00013

[24] Qu J, Li S, Yu D. Detection of complex chromosome rearrangements using optical genome mapping. Gene. 2023;884(30):147688. 37543218 10.1016/j.gene.2023.147688

